# The publication of ethically uncertain research: attitudes and practices of journal editors

**DOI:** 10.1186/1472-6939-13-4

**Published:** 2012-04-11

**Authors:** Carla Angelski, Conrad V Fernandez, Charles Weijer, Jun Gao

**Affiliations:** 1 Division of Pediatric Emergency Medicine, University of Alberta, Edmonton, AB, Canada; 2 Department of Hematology and Oncology, Dalhousie University, Halifax, NS, Canada; 3 Roman Institute of Philosophy, Western University, London, ON, Canada; 4 Surveillance and Epidemiology Unit, Cancer Care Nova Scotia, Halifax, NS, Canada; 5 Stollery Children's Hospital, Edmonton Clinic Health Academy, 11405-87 Ave, Edmonton AB, T6G 1C9, Canada

**Keywords:** Editors, Ethics, Publication ethics

## Abstract

**Background:**

Publication of ethically uncertain research occurs despite well-published guidelines set forth in documents such as the Declaration of Helsinki. Such guidelines exist to aide editorial staff in making decisions regarding ethical acceptability of manuscripts submitted for publication, yet examples of ethically suspect and uncertain publication exist. Our objective was to survey journal editors regarding practices and attitudes surrounding such dilemmas.

**Methods:**

The Editor-in-chief of each of the 103 English-language journals from the 2005 Abridged Index Medicus list publishing original research were asked to complete a survey sent to them by email between September-December 2007.

**Results:**

A response rate of 33% (n = 34) was obtained from the survey. 18% (n = 6) of respondents had published ethically uncertain or suspect research within the last 10 years. 85% (n = 29) of respondents stated they would always reject ethically uncertain articles submitted for publication on ethical grounds alone. 12% (n = 4) of respondents stated they would approach each submission on a case-by-case basis. 3% (n = 1) stated they would be likely to publish such research, but only with accompanying editorial. Only 38% (n = 13) give reviewers explicit instruction to reject submissions on ethical grounds if found wanting.

**Conclusions:**

Editorial compliance with the Declaration of Helsinki in rejecting research that is conducted unethically was difficult to ascertain because of a poor response rate despite multiple attempts using different modalities. Of those who did respond, the majority do reject ethically suspect research but few explicitly advise reviewers to do so. In this study editors did not take advantage of the opportunity to describe their support for the rejection of the publication of unethical research.

## Background

The first formal request to editors of biomedical journals by the American Medical Association in 1912 asked them to "scrutinize carefully original papers submitted for publication" in the context of compliance with principles of medical ethics [1,2]. The Declaration of Helsinki (DoH) in 1964 specifically addressed the issue of publication when research is found lacking in any or all of its recommended tenets; "both authors and publishers have ethical obligations...reports of experimentation not in accordance with the principles laid down in this declaration should not be accepted for publication"[3,4]. Despite these regulations, attitudes and practices surrounding the publication of ethically suspect research is relatively understudied.

Though most editors and journals follow the tenets of the DoH, publication of unethical research still occurs [5,6]. Why its principles are not consistently complied with is unclear. There is argument that not allowing for flexibility in publication of ethically suspect research does a disservice to both human participants and the scientific community [7]. Some argue that non-publication engenders an unrealistic perception that unethical research is not occurring [8]. Some biomedical journals have chosen to publish ethically suspect research with an accompanying editorial in hopes of fostering discussion of ethical challenges [9,10]. This is particularly relevant when the issue is not clearly a breach of established ethical conduct. Further argument for publication of ethically suspect research lies in the potential loss of valuable scientific information, the risks assumed by the research participants and the need to expose further research participants to risk if the research is to be replicated[8].

In 1977, a survey of 138 core medical journal editors was administered by Brackbill and Hellegers regarding whether or not they would personally or professionally publish unethical research. The survey results were limited by a low response rate but indicated an apparent consensus of opinion concerning editorial responsibility to screen for and publish ethical research. This survey predated the formation of agencies such as the International Commission of Medical Journal Editors (ICMJE), which sets forth ethical guidelines regarding publication [11]. In 1977 only a minority of journals required authors to submit details regarding obtaining informed consent or research ethics board (REB) approval and the majority of editors did not instruct the reviewers to consider ethical issues in their assessments of submitted manuscripts [12,13]. Our primary objective, therefore, was to survey editors of all 103 English language journals in the Abridged Index Medicus. We hoped to describe modern era attitudes and practices with respect to the publication of ethically suspect research.

Our hypotheses were: 1) Few journal editors would accept ethically uncertain research for publication today, 2) Of those publishing ethically uncertain research, the majority would indicate they would supply an accompanying editorial, 3) Of those refusing to publish ethically uncertain research a higher number of editors would instruct their reviewers to screen for ethical violations, and finally that 4) Editors would be more likely to publish individual case reports without meeting full ethical requirements than clinical trials. We describe these results and the implications of a relatively low response rate with respect to the responsibility of editors to transparently demonstrate compliance with the Declaration of Helsinki.

## Methods

All components of the study requiring contact with human participants were approved by the IWK Health Centre REB committee. The Editor-in-chief of each of the 103 English-language journals from the 2005 Abridged Index Medicus list (last updated February 2005) publishing original research were asked to complete a survey sent to them by email between September-December 2007. If the initial email was unsuccessful, the non-responders were contacted by phone to ensure correct electronic address and the survey was resent. Email addresses and phone numbers for editorial offices were attained via publicly available resources. The Editor-in-chief was instructed to forward the survey to an associate editor if (s)he felt it more appropriate.

The 25 item survey was designed using the method of Dillman[14]. It included a demographic section to describe the population of respondents (see Table 1). Questions were derived from a review of the literature including the previous Brackbill survey in 1977 [12]. Our aim was to identify if and under what circumstances editors are willing to publish ethically uncertain research. We also wanted to identify what editors perceived as "ethically uncertain". Prior to administration, the survey underwent a content validity exercise with 8 experts in editorial work and research publication and the survey modified accordingly. We also piloted the survey with 8 physicians who had editorial and publication experience for length and readability. An optional text box concluded the survey to allow respondents a location to indicate whether they would like a copy of the research results and also be included in our book prize draw, which was used as an incentive to encourage response. This information was kept confidential and separate from the data. Statistical analysis was by descriptive methods of frequency. Chi square analysis was planned, with view to apply the Fisher Exact test if sparse cells existed, but not conducted given our low response rate.

**Table 1:** Demographics of respondents

Type of Journal:	(n)	(%)
*General Medical*	11	32
*Subspecialty*	23	68
Age Group:	10	29
*46-55 y*	24	71
*> 55 y*		

Gender:	5	15
*Female*	29	85
*Male*		

Country Main Editorial Office:	2	6
*Canada*	28	82
*USA*	1	3
*UK*	2	6
*India*	1	3
*Other*		

Editorial Position Head:	32	94
*Editor-In-Chief*	2	6
*Other*		

Length of time in Journal:	14	41
*1-3 y*	11	32
*4-6 y* *> 7 y*	9	27

# of Journals You Have Edited:	16	47
*1-3*	9	26.5
*4-6*	9	26.5
*> 7 y*		

Education Held:	28	82
*MD*	8	23.5
*PhD*	4	12
*Formal Bioethics Training*	1	3
*ICMJE*	4	9
*Other*		

Country of Training:	29	85
*USA*	3	9
*Canada*	2	6
*Other*		

Formal Bioethics Training:	13	38
*Yes*	21	62
*No*		

## Results

The response rate for completion of our survey was 33% (n = 34). 6 potential respondents declined due to time constraint. 11 potential respondents had out of date contact information or unpublished email or phone numbers. 52 potential respondents did not reply. Repeated efforts were made to contact these potential respondents who did not reply via email and telephone over a 1 year period. Demographic characteristics of the respondents are shown in Table 1.

When polled as to whether the editor had published ethically uncertain research during their tenure as editor; 15% (n = 5) replied, "yes", while 85% (n = 29) said "no". 85% (n = 29) of respondents stated that they would always reject ethically uncertain articles submitted for publication on ethical grounds alone. 3% (n = 1) indicated they would accept ethically uncertain research for publication under certain circumstances but always with an editorial to identify ethical issues. 12% (n = 4) of respondents stated they would approach each submission on a case-by-case basis and often refer questionable manuscripts and their authors back to the Declaration of Helsinki. They also indicated that occasionally they then would allow resubmission if identified ethical issues had been satisfactorily addressed.

A Likert scale was used to identify primary reasons editorial staff may consider publishing ethically uncertain research (see Table 2). Reasons provided to reject publication of ethically uncertain research were explored; respondents could select more than one reason. 82% (n = 28) of respondents either agreed or strongly agreed that publishing ethically uncertain research would not be in accordance with international standards. 79% (n = 27) agreed or strongly agreed that they would not publish such research in order to deter its future submission. 85% (n = 29) felt they had a responsibility to readers not to publish ethically uncertain research. 62% (n = 21) felt that the quality of the science would also be suspect when reviewing ethically uncertain research submitted for publication.

**Table 2:** Degree to which each respondent agreed with each statement

Re: Publishing Ethically Uncertain Research n = 34	Strongly Disagree (%)	Disagree (%)	Agree (%)	Strongly Agree (%)
Would publish if value in data collected	21(62)	7(21)	4(12)	2(6)

Would publish to make it visible	25(74)	5(15)	3(9)	1(3)

Would publish for educatinal value of Collective Scrutiny	23(68)	4(12)	5(15)	2(6)

Would publish so risk to participants not recurrent	20(59)	9(26)	1(3)	4(12)

Would publish as some areas "grey" and warrant discussion	12(35)	11(32)	9(26)	2(6)

The editors were then asked how often, within the last 5 years, they had: i) become aware of ethically uncertain research upon reviewing manuscripts for publication ii) Informed the author's parent institution of submission of ethically uncertain research, and iii) Informed the author's funding agency of ethically uncertain research if submitted. The results can be found in Table 3. The respondents indicated that the infractions most likely to cause them to notify institutions or funding agencies were: fraudulent data (88%; n = 30), duplicate publication (68%; n = 23), conduct without REB approval (50%; n = 17), lack of consent obtained (50%; n = 17), "other" breaches of the Declaration of Helsinki (50%; n = 17) and disproportionate risks to research participants (44%; n = 15). Only 12% (n = 4) indicated they would not inform either the funding agency or the parent institution of ethical infractions noted upon review.

**Table 3:** Frequency of presentation of different scenarios

In the past 5 y, how often has your journal: n = 34	0 (%)	1-3 (%)	4-7 (%)	8-10 (%)	> 10 (%)
1. Became aware of ethically uncertain research upon rev. manuscript for publication?	5(15)	14(41)	8(24)	6(18)	1(3)

2. Informs the author's parents inst. of submission of ethically uncertain research?	13(38)	14(41)	6(18)	1(3)	0(0)

3. Informed the author's funding agency of ethically uncertain research if submitted?	29(85)	3(9)	1(3)	1(3)	0(0)

The majority of respondents indicated that they would review various study designs with the same or greater stringency when compared with that used to review a phase III trial design (see Table 4). Editors were asked what they viewed their primary role as; that of "rule-maker", "gate-keeper" or to promote discussion surrounding ethically uncertain issues. 61% (n = 21) agreed or strongly agreed that their role included that of "rule-maker". 89% (n = 31) agreed or strongly agreed that their role included that of "gate-keeper". 76% (n = 26) believed they had a role to play in promoting discussion surrounding ethically uncertain issues.

**Table 4:** Degree of stringency to which respondents review the following types of manuscripts submitted compared with a phase III trial

n = 34	More (%)	Same (%)	Less (%)
Phase I	3(9)	25(75)	6(18)

Phase II	3(9)	28(82)	3(9)

Phase IV	2(6)	30(88)	2(6)

Cohort	1(3)	26(76)	7(21)

Secondary Use of Data	2(6)	21(32)	11(32)

Survey	3(9)	18(53)	13(38)

Case Report	3(9)	15(54)	16(47)

When asked if their journal instructed reviewers to examine submitted manuscripts for adherence to applicable ethical regulatory guidelines, 70% (n = 24) indicated they did. When asked if their journal instructed reviewers to recommend rejection of a submitted manuscript on the basis of lack of adherence to applicable ethical regulatory guidelines, 62% (n = 21) indicated they did not. We asked editors to describe an incident of unethical research to give an overview of common challenges. Almost all editors, 79% (n = 27), replied with examples of specific events with the majority citing duplicate publication or plagiarism and to a lesser degree risk to participants, justice issues and fabrication of data. Many editors noted that they not only addressed this with the author directly but also notified responsible institutions.

## Discussion

Our study's low response rate inhibits our ability to draw robust conclusions. It demonstrates in the respondents a high degree of consensus that editors would comply with the Declaration of Helsinki in rejecting publication of ethically suspect research submitted for review. This would seem to be consistent with the findings of Brackbill and Hellegers published 30 years ago but is similarly limited to by a relatively poor response rate. We have attempted to identify the rationale given by editors in considering rejection of ethically suspect research. Those editors that did respond indicated that deterrence to future submission of unethical research, a desire to comply with international regulations and a feeling of responsibility to the public and scientific community all play a roll in their decision to reject such manuscripts.

Given the high degree that the responding editors indicated they would not publish ethically suspect research, one might expect that nearly all of the respondents and their journals would specifically instruct reviewers to examine submitted manuscripts for adherence to applicable ethical regulatory guidelines and indeed most do. We have corroborated this finding in a separate study that directly examined instructions to authors [15]. We further noted that just 38% (n = 13) of journals would specifically instruct their reviewers to reject manuscripts based on ethical grounds alone. Again, we cannot draw a definitive conclusion but believe this may be a lost opportunity to proactively harness the strength of the scientific community in assessing and recommending rejection of unethical research. This could be rectified with relatively simple additions within the instructions to reviewers.

In an editorial in the British Journal of Medicine in 2004, Fiona Godlee states that "editors have no mandate" to govern their conduct [16]. Although agencies such as the Committee on Publication Ethics (COPE) and the World Association of Medical Editors (WAME) have attempted to publish codes of conduct for editors specifically, breeches of them are unenforceable as they have no legal jurisdiction [17-19]. Such codes have been in existence long before we conducted our survey. Should we be encouraging mandatory membership of all medical journals to a regulatory body that holds each accountable? Or does this dissuade the high degree of academic freedom that many editors enjoy?

Although editorial adherence to guidelines is encouraged and even expected, it remains variable how such codes are interpreted and implemented. Godlee as one of the founders of the first iterations of COPE guidelines, states that editors may be tempted to simply refuse to publish a paper based on "other" (presumably *ethical) *grounds alone [17]. COPE mandates that this alone, is not appropriate and states that editors should investigate all serious allegations fully so as editors take their role as "guardians" of the biomedical literature seriously [17]. Of interest, the majority of editors in our survey will not only reject a paper on ethical grounds but also take measures to inform the host institution and/or funding agencies. This more specifically adheres to the ICMJE, COPE and WAME recommendations on how to approach such submitted research.

Editorial obligation is extensive. Do they have time to act as "responsible social agents" and hold authors accountable in all cases for possible ethical infractions [19]? Once research misconduct is identified one may argue that handing over responsibility to an agency whose mandate it would be to deal with such allegations would be a more efficient model. This may be reflected by the 61% (n = 21) of respondents in our survey who viewed themselves as "rule-makers" versus the higher proportion of 89% (n = 31) who viewed themselves as "gate-keepers". By defining editorial obligation in publication, aforementioned agencies such as COPE, ICMJE and WAME are serving as the foundation upon which renewed calls to editors are sounded. There have been efforts to re-examine consequences, such as impact upon membership, by members who do not comply with these guidelines [17]. Does this constrain editorial freedom or ensure scientific accountability?

Limitations of our study include a relatively small response rate. Although average response rates of email surveys have been quoted at roughly 30%, we expected a much higher return given the study content and sample population [14]. It is distressing that editors did not take the opportunity to publically share their editorial practice in a transparent way. This we feel was a lost opportunity to add transparency to the editorial process of scientific review and therefore underpin public confidence in the research enterprise. We theorize that because editors often have many demands on their time, the emailed survey may have been overlooked or deleted without further attention. Given the importance of the ethical questions raised, one should consider the implications such dismissal has on what is indeed published within the medical literature. Is ethically suspect or concerning material dissected appropriately at all times?

Attempts to rectify our response rate were made by contacting editorial offices directly by telephone. This was also a challenge as many editors would either not respond to the call or fail to return our repeated attempts at contacting them. We considered that non-respondents were hesitant to participate in such a survey given potential responses having a high degree of visibility within the bioethical community. This seems unlikely given the number of respondents that consider themselves 'gatekeepers' of the literature. The possibility that responses were biased by social and editorial acceptability, which may not reflect actual practice, may have also impacted the results we obtained. We feel this is unlikely however, given the confidential nature of the survey, a typically strong sense of academic freedom in individuals charged as editors and that editors-in-chief intrinsically have positions that require a high degree of integrity and accountability. Although there was editorial representation from a variety of specialty and general medical journals publishing original human research, generalizability was compromised given our sample size.

## Conclusions

In conclusion, we have found that responding editors are reasonably consistent and compliant with the Declaration of Helsinki in rejecting publication of ethically uncertain research. Editors seem to identify a mixed understanding of their roles in research misconduct oversight but for the most part play a strong role in rejection of ethically suspect research and in notifying appropriate institutions. Opportunity exists to strengthen review of ethically suspect research by more deliberately charging reviewers with the task of both reviewing manuscripts with respect to compliance of ethical tenets, and rejecting those that do not meet applicable international standards. Editors should consider adopting uniform practices when they do publish ethically suspect research such as alerts to the reader and/or editorial comment. Finally, the scientific community must continue to define whom they are accountable to so that ethical infractions continue to be dissuaded.

## Competing interests

The authors declare that they have no competing interests.

## Authors' contributions

CA was involved in the conception, design, conduction of the project as well as manuscript preparation and revisions. CVF was involved in the design of the project as well as manuscript revision. CW was involved in manuscript revision. JG was involved in statistical conception and manuscript revision. CA, CVF and CW participated in final manuscript revision.

## Funding source

IWK Category "A" Grant

## Pre-publication history

The pre-publication history for this paper can be accessed here:

http://www.biomedcentral.com/1472-6939/13/4/prepub
